# Supplementary material for: Flexible TAM requirement of TnpB enables efficient single-nucleotide editing with expanded targeting scope
Source: Nat Commun. 2024 Apr 24;15:3464. doi: 10.1038/s41467-024-47697-4 (PMC11043419; doi:10.1038/s41467-024-47697-4)

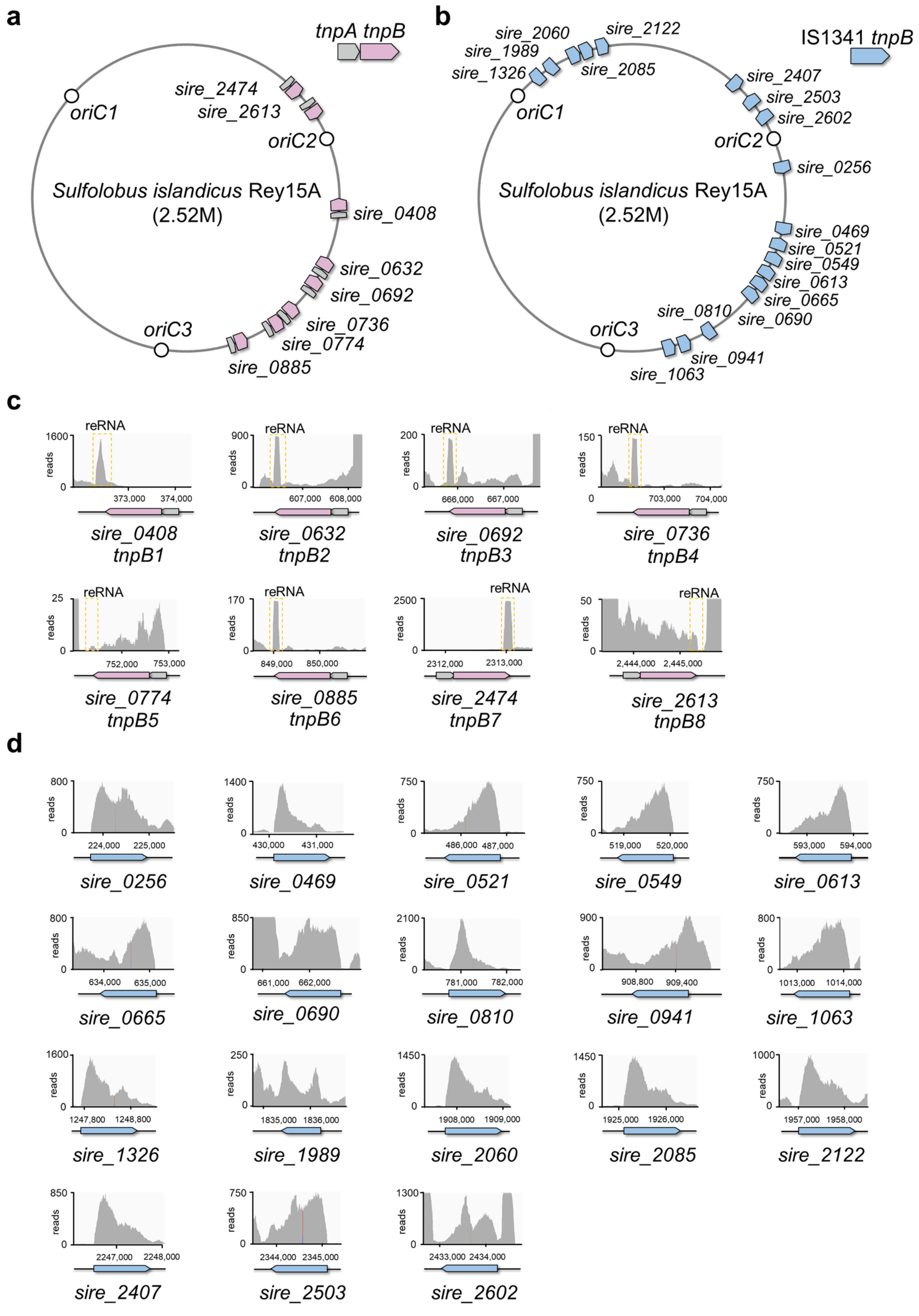

Supplementary Figure 1. TnpBs of IS605 and IS1341 types encoded by *Sulfolobus islandicus* REY15A

Sulfolobus islandicus encodes 8 IS605-type TnpBs (a) and 18 IS1341-type TnpBs (b). The circles indicate replication origins. The transcriptome data were retrieved from our published transcriptome data [1] and reads coverage was visualized with IGV (Integrative Genomics Viewer) [2] (c for IS605 *tnpB*s and d for IS1341 *tnpB*s).

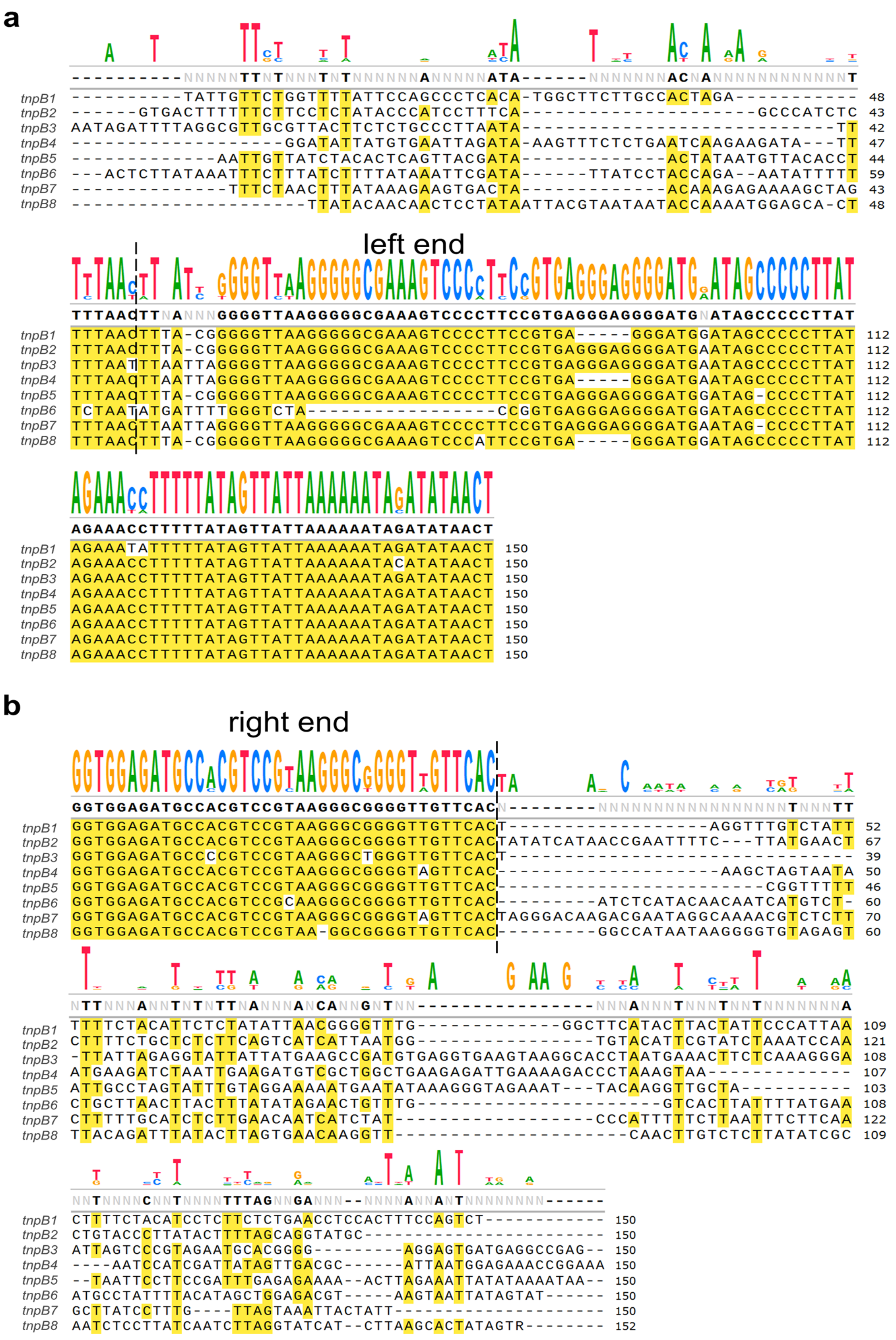

Supplementary Figure 2. Multiple sequence alignment of IS200/IS605-family transposon and its flanking sequences

(a) Multiple sequence alignment of 150 nt sequences preceding the start codon (ATG) of 8 *tnpA* genes of the IS605 transposons in *S. islandicus* REY15A. (b) Multiple sequence alignment of 150 nucleotides downstream of stop codon (TGA) of 8 *tnpB*s of the IS605 transposons. Sequences are aligned using the MUSCLE algorithm and visualized using the SnapGene.

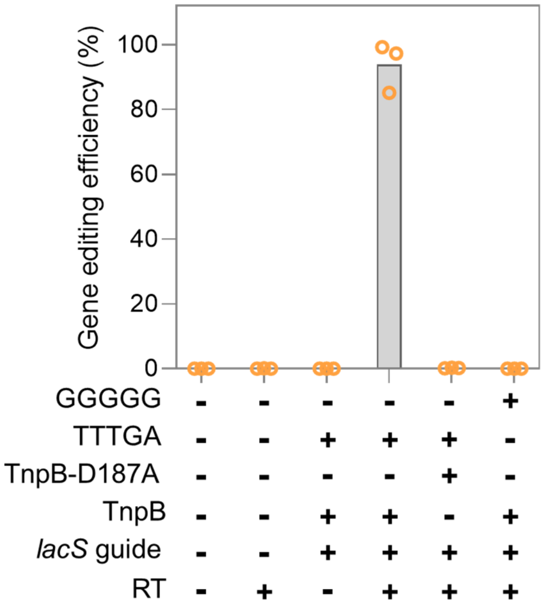

Supplementary Figure 3. Determinants of gene editing in *Sulfolobus* with TnpB7
The g(1)*lacS*-RT gene editing plasmid targeting the genomic *lacS* gene was used to investigate the determinants of gene deletion with TnpB. TnpB-D187A means the catalytic dead version of TnpB7. GGGGG and TTTGA refer to strains in which the 5' flanking sequences of the target region (NTS strand) are GGGGG and TTTGA, respectively. RT refers to the repair template. Bars represent means of three biologically independent experiments. Source data are provided as a Source Data file.

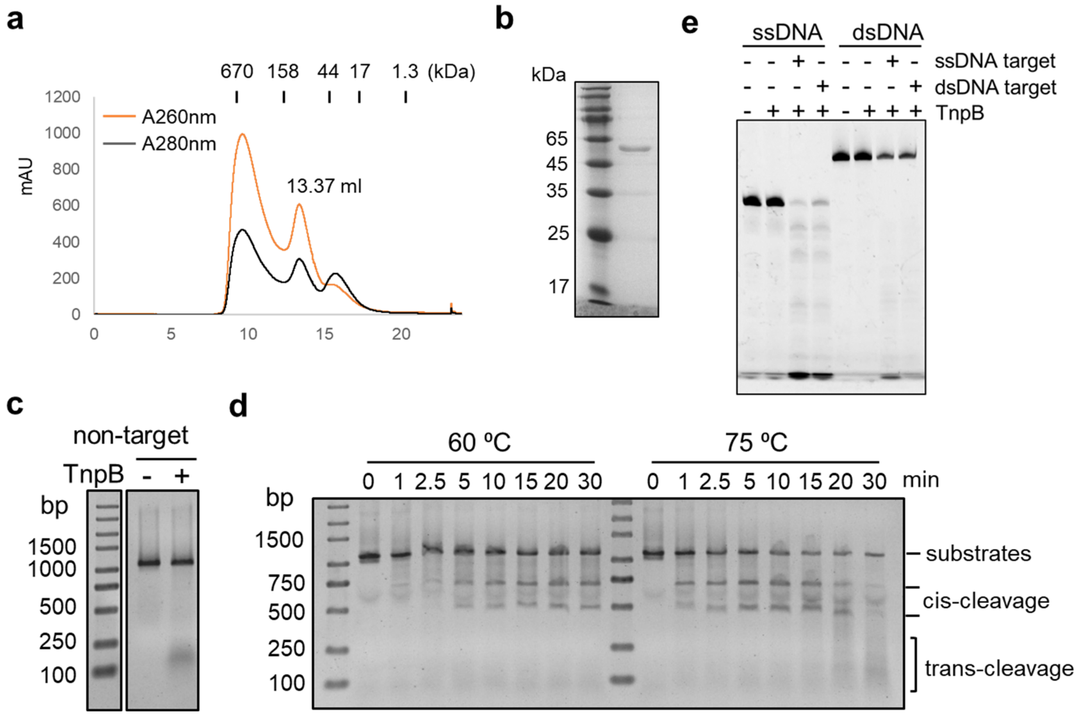

Supplementary Figure 4. SisTnpB7 forms RNA associated protein complex that cleaves target DNA

(a) Gel filtration analysis of TnpB RNP complex eluted from Histrap column. The elution volume of the protein standard is indicated. Proteins eluted at 13.37 ml were combined and used for subsequent assays. (b) SDS_PAGE analysis of the TnpB RNP. (c) TnpB does not cleave unspecific dsDNA. The non-target refers to the dsDNA substrate that does not contain the *lacS* target region. (d) dsDNA target cleavage by TnpB at 60 ºC or 75 ºC. The reaction system consists of 20 nM dsDNA substrates and 200 nM TnpB RNP. Reactions were incubated for 0, 1, 2.5, 5, 10, 15, 20, 30 min. (e) TnpB7 shows trans cleavage activities upon the presence of target. The ssDNA substrate and dsDNA substrates are labeled by 5’ FAM. 50 nM activator DNA (ssDNA target or dsDNA target), substrates and 300 nM TnpB RNP were used and the reactions were incubated at 75 ºC for 10 min.

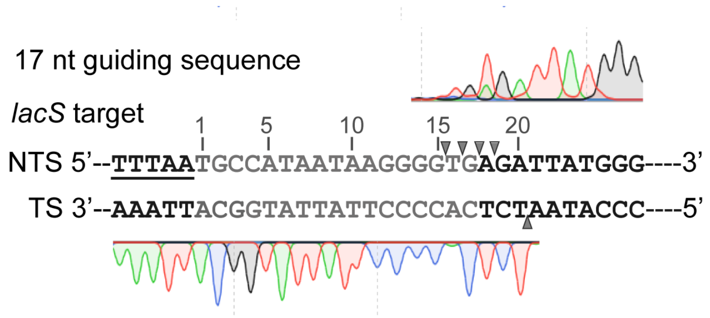

Supplementary Figure 5. Cleavage pattern of TnpB7 with a 17 nt guiding sequence

Cleavage positions at the non-targeted strand (NTS) and the target strand (TS) are indicated by grey triangles. The sequences of the guide-target duplex region are shown in grey and the TAM sequence is underlined. 200 nM TnpB RNP and 2 µg linear dsDNA substrates were incubated at 70 ºC for 60 min and the reaction products were sent to run-off sequencing. Sanger sequencing results of the cleavage products were used to define the cleavage sites shown in the figure.

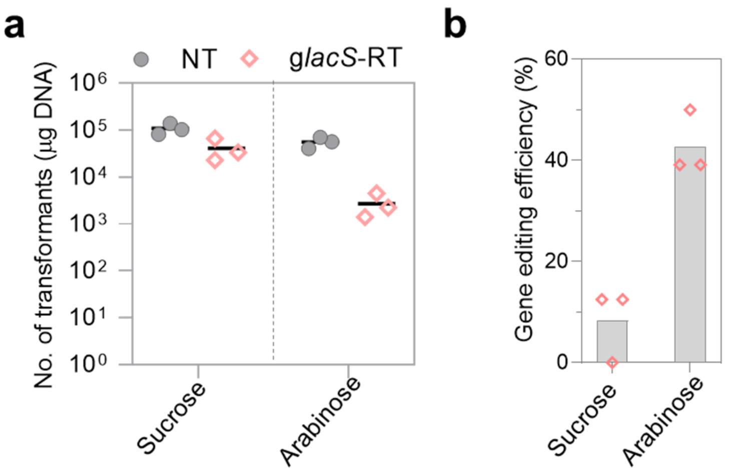

Supplementary Figure 6. Gene editing assay on the TCTGA weak TAM with TnpB7 of different expression strengths

(a) Transformation efficiency with the NT and *lacS*-editing plasmid (g*lacS*-RT) determined using media containing 0.2% Sucrose or D-arabinose. The strain S7 containing the *lacS* target flanked by the TCTGA TAM was used for the transformation assay. Data are obtained from three biologically independent experiments. (b) Gene editing activities of TnpB7 under the basal expression level (Sucrose) and the induced condition (Arabinose). Bars represent means of three biologically independent experiments. Source data are provided as a Source Data file.

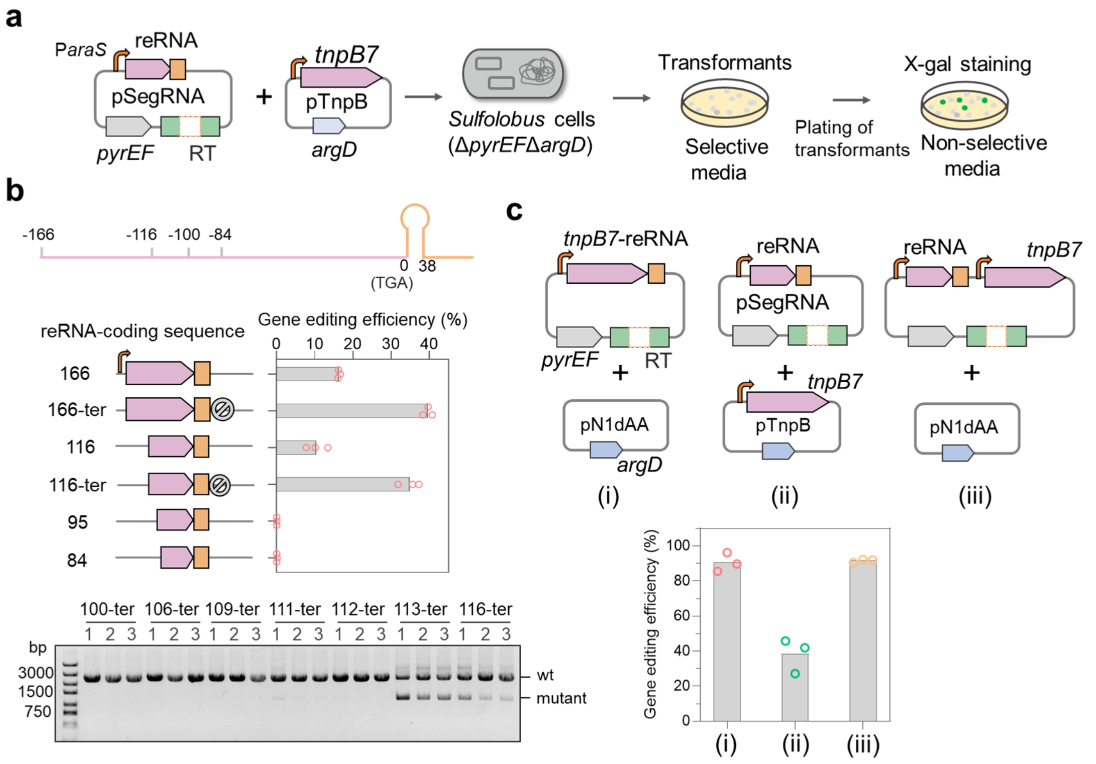

Supplementary Figure 7. Characterizations of reRNA required for gene editing (a) A workflow of gene editing experiment using the dual-plasmid gene editing system. (b) Gene editing efficiencies of guide RNA scaffolds of different length. 166, 116, 113, 112, 111, 109, 106, 100, 95 and 84 indicate the length of the CDS-derived RNA and they all share the same 3' terminal conserved right end (38 nt) and a guide (25 nt) targeting the genomic *lacS* gene. 166-ter contains a transcriptional terminator sequence immediately downstream of the *lacS* guiding sequence. 1, 2, 3 indicate three replicate experiments. PCR genotyping was performed with 10 randomly selected transformants as templates. (c) Gene editing activities of different plasmid combinations. pN1ddA is an empty vector used for the construction of pTnpB plasmid. Data are obtained from three biologically independent experiments. Source data are provided as a Source Data file.

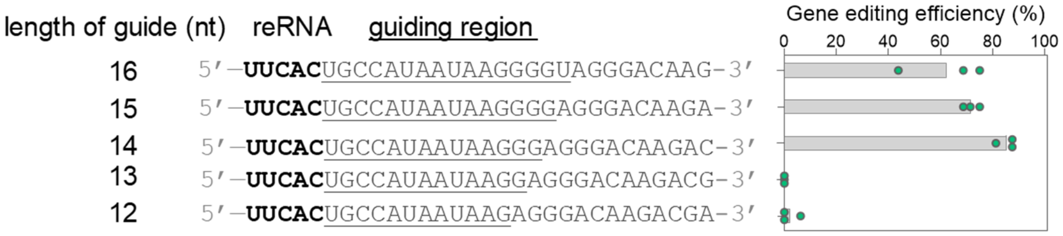

Supplementary Figure 8. Guide length requirement upon a GTTCA TAM
The minimal length of the *lacS* guide sequence that supports gene editing activity of TnpB on the target flanked by GTTCA TAM. The guide-target duplex regions are underlined. Data are obtained from three biologically independent experiments. Source data are provided as a Source Data file.

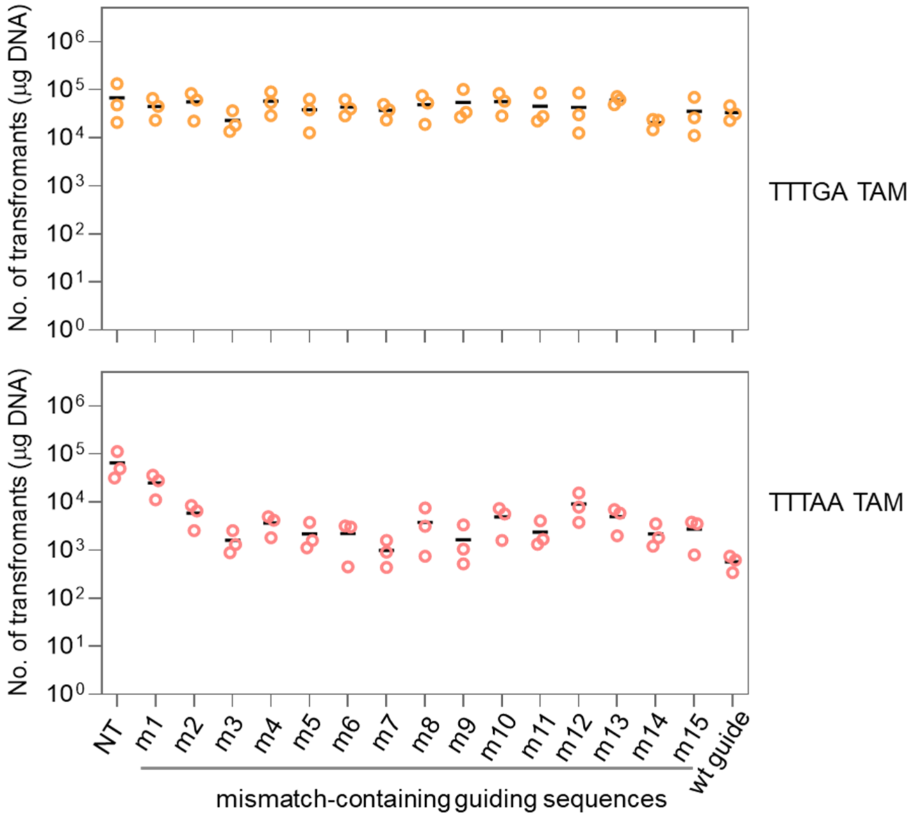

Supplementary Figure 9. Transformation efficiency with gene editing plasmid containing mismatched guiding sequences upon strong and weak TAMs

NT means non-targeting plasmid. m1 to m15 means the *lacS* gene editing plasmid expressing mismatched guide RNA. wt guide means the *lacS*-editing plasmid expressing the full-matching guide RNA. The transformation efficiency was defined as the colony formation unit of electroporated cells per 1 µg plasmid DNA. The line indicates the mean of three biologically independent experiments. Source data are provided as a Source Data file.

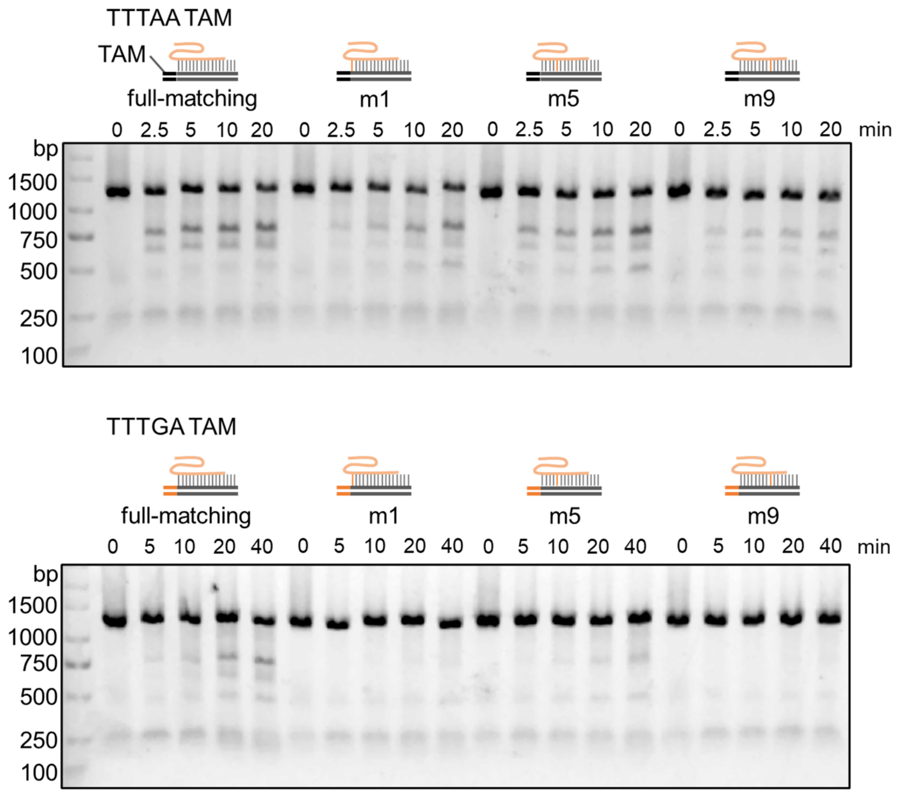

Supplementary Figure 10. DNA cleavage activities of TnpB on mismatched targets flanked by TTTAA or TTTGA

Time-resolved target or mismatched target DNA cleavage with TnpB RNP. The reaction consists of 200 nM TnpB RNP, 40 nM dsNA substrate (1120 bp) containing the 25 nt *lacS* target region and was incubated at 70 ºC for indicated period (0, 2.5, 5, 10, and 20 min for the TTTAA substrate; 0, 5, 10, 20 and 40 min for the TTTGA substrate). All experiments were repeated three times independently with similar results.

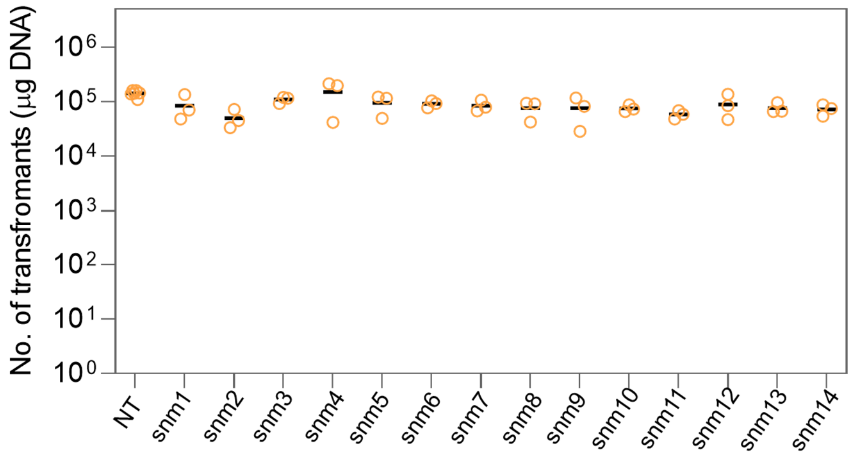

Supplementary Figure 11. Genome targeting assay with SNE plasmids upon the TTTGA TAM

NT means non-targeting plasmid. snm1 to snm14 mean the SNE plasmids targeting the genomic *lacS* target but containing different RTs. The transformation efficiency was defined as the colony formation unit of electroporated cells per 1 µg plasmid DNA. The line indicates the mean of three biologically independent experiments. Source data are provided as a Source Data file.

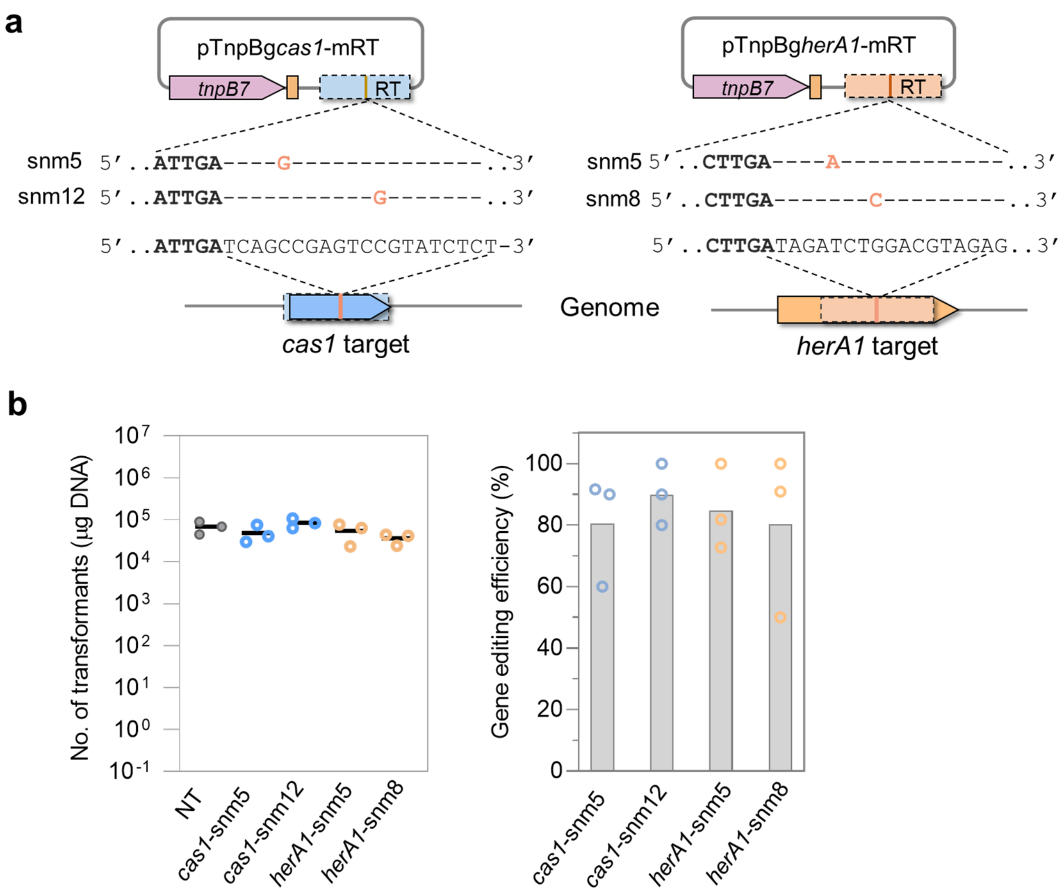

Supplementary Figure 12. Gene editing assay targeting *herA1* and *cas1* genes to introduce single-nucleotide mutations

(a) A diagram of the SNE plasmids used for the generation of single-nucleotide mutagenesis at *cas1* and *herA1* target sites flanked by ATTGA and CTTGA respectively. Mismatched positions between the guide RNA and repair templates are 5th and 12th for the *cas1* target, and 5th and 8th for the *herA1* target. RT means repair template. (b) Gene editing outcomes on these two target genes with TnpB. NT refers to a non-targeting control plasmid. The genotypes of edited colonies were confirmed by the sanger sequencing of PCR amplicon of the target site. Bars indicate the means of three biologically independent experiments. Source data are provided as a Source Data file.

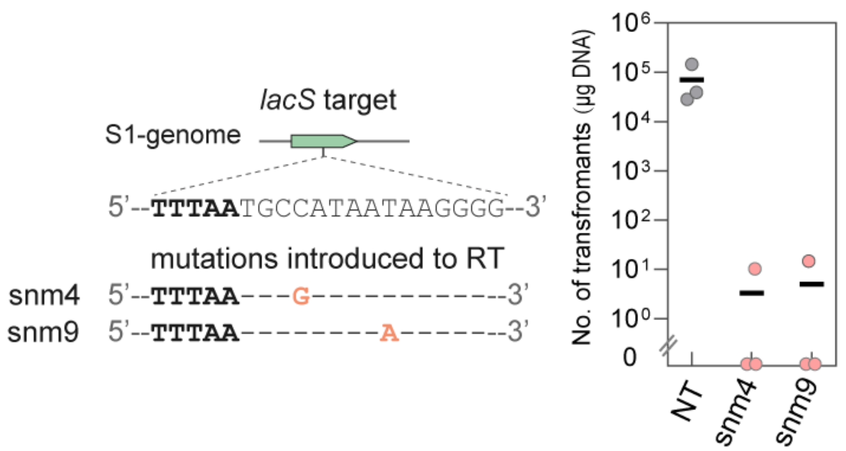

Supplementary Figure 13. Gene-editing assay with SNE plasmids on the *lacS* target flanked by the TTTAA TAM.

snm4 and snm9 refer to the SNE plasmids carrying target-mismatched RT. NT means the non-targeting control. Lines in the graph indicate the mean of three independent experiments. Source data are provided as a Source Data file.

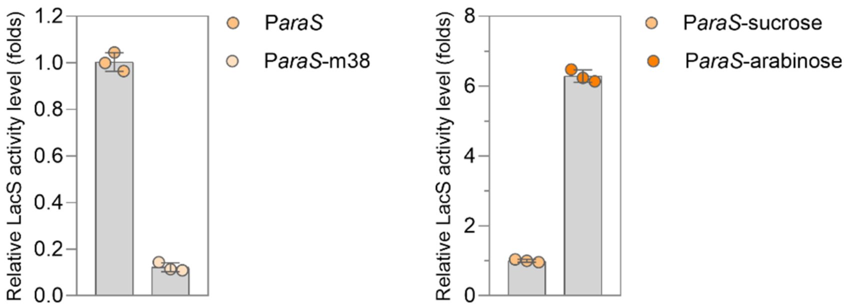

Supplementary Figure 14. The relative strength of different promoters as determined by the galactosidase assay

(a) The relative strength of the P*araS*-m38 promoter in driving the gene expression LacS was determined by measuring the galactosidase activity of the LacS protein. The activities were determined under the basal expression level (sucrose). The activity of the original promoter was defined as 1.0, with which the activity of P*araS*-m38 was calculated. (b) The expression strength of the P*araS* promoter with the media containing sucrose (basal expression) or arabinose (induced condition). The activity of the P*araS* promoter under the sucrose condition (P*araS*-sucrose) was defined as 1.0. Bars indicate the mean of three independent experiments. Source data are provided as a Source Data file.

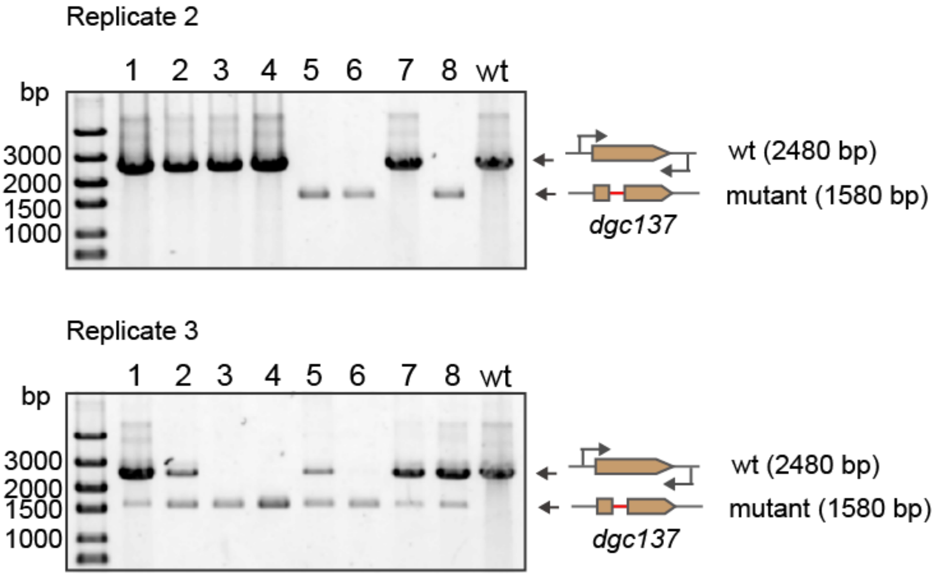

Supplementary Figure 15. TnpB facilitates gene deletion in *Vibrio alginolyticus*
PCR genotyping results of two replicate experiments related to Figure 6c. For each replicate experiment, 8 randomly selected colonies from the streak plate were used as the templates for the PCR genotyping with the check primers. wt, wild type.

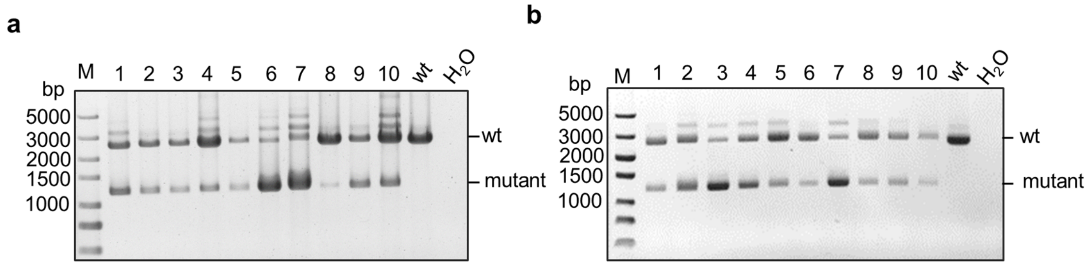

Supplementary Figure 16. Mixed genotypes of *Sulfolobus* transformants

10 randomly selected colonies from the transformants plate (a for TTTAA TAM and b for TTTGA TAM) were used as the templates for PCR genotyping. M, DNA marker; 1-10, 10 randomly selected transformants. wt, wild type control. The size of expected PCR products is 2.5 kb for the wt and 1.2 kb for the gene-deletion mutant.

Supplementary Table 1. A summary of TAM variant sequences for TnpB gene editing

In vitro activity[a] (substrate cleaved %)	TAM sequences[b]	Sequence logo	Gene editing outcomes[c]	
27-48% (12 TAMs)	**TTTAA**, **GTTAA**, **ATTAA**, **CTTAA**, **TTTCA**, **TATAA**, **GATAA**, AATAA, CATAA, **GTTCA**, ATTCA, CTTCA	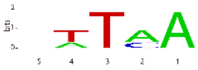 (>27%)	Cell death for gene-targeting plasmid and gene editing plasmid	Efficient gene deletion, SNE editing requires reducing the expression of TnpB.
18.5-27% (10 TAMs)	**TCTAA**, ACTAA, CCTAA, GCTAA, **TGTAA**, AGTAA, CGTAA, GGTAA, **TTTTA**, **TTTAT**	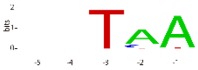 (>18.5%)	Not tested	Not tested, Predicted to be efficient in gene editing
11.5-18.5% (10 TAMs)	**TTTGA**, **TTTAC**, **TTAAA**, **GTTTA**, ATTTA, CTTTA, **GTTGA**, ATTGA, CTTGA, **TTTAG**	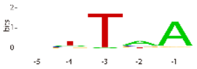 (>11.5%)	No cell death for either gene-targeting plasmid or gene editing plasmid	Efficient gene deletion and SNE.
4-11.5% (24 TAMs)	**GTTAC**, ATTAC, CTTAC, GTTAT, ATTAT, CTTAT, GTTAG, ATTAG, CTTAG, **GTAAA**, ATAAA, CTAAA, **TAAAA**, TGAAA, TCAAA, **TATTA**, **TATCA**, **TATGA**, TGTGA, TGTTA, TGTCA, **TCTGA**, TCTTA, TCTCA	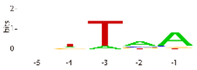 (>4%)	No cell death for either gene-targeting plasmid or gene editing plasmid	Efficient gene editing requires elavating the expression of TnpB.

a. The in vitro activities of different TAM variants were determined in Figure 2.
b. Since TnpB showed no preference on all other three mutated nucleotides at -1, -4 and -5 position, TAM variant sequences with similar activities compared to characterized TAM sequences (highlighted by bold letters) can be inferred.
c. The gene editing outcomes are based on the basal expression level (P*araS* in the presence of 0.2% sucrose) as defined in this study. Variations in the expression strength of TnpB or reRNA may affect the gene editing outcomes. Sequence logos were generated with WebLogo[3]

References

1 Sun, M. *et al.* An Orc1/Cdc6 ortholog functions as a key regulator in the DNA damage response in Archaea. *Nucleic Acids Res* **46**, 6697-6711, doi:10.1093/nar/gky487 (2018).

2 Thorvaldsdottir, H., Robinson, J. T. & Mesirov, J. P. Integrative Genomics Viewer (IGV): high-performance genomics data visualization and exploration. *Brief Bioinform* **14**, 178-192, doi:10.1093/bib/bbs017 (2013).

3 Crooks, G. E., Hon, G., Chandonia, J. M. & Brenner, S. E. WebLogo: a sequence logo generator. *Genome research* **14**, 1188-1190, doi:10.1101/gr.849004 (2004).

Source Data- Supplementary Figure 7b

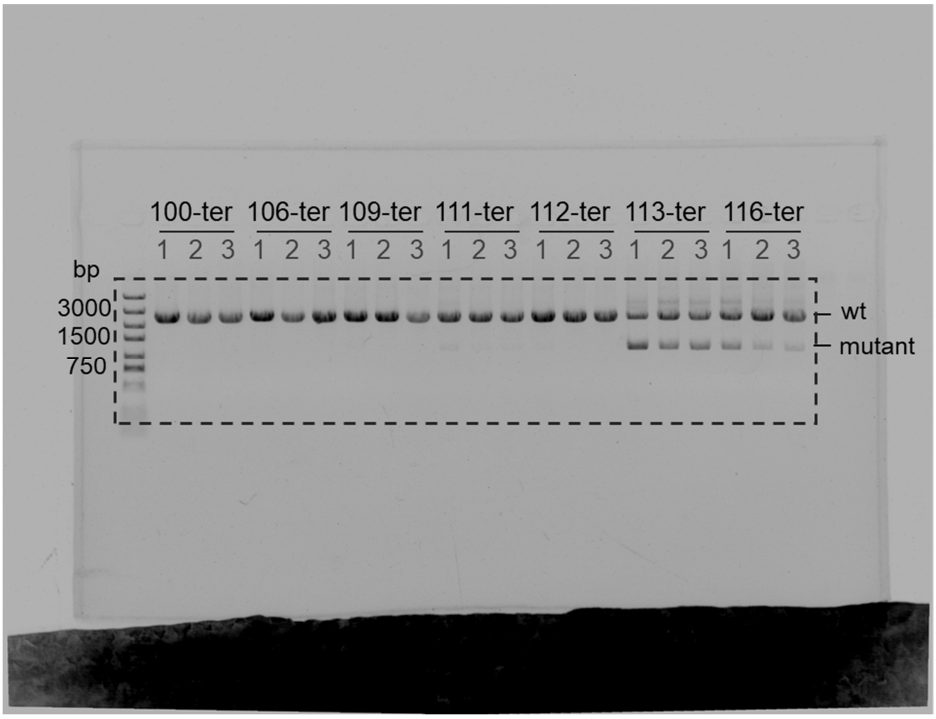

Source Data- Supplementary Figure 10

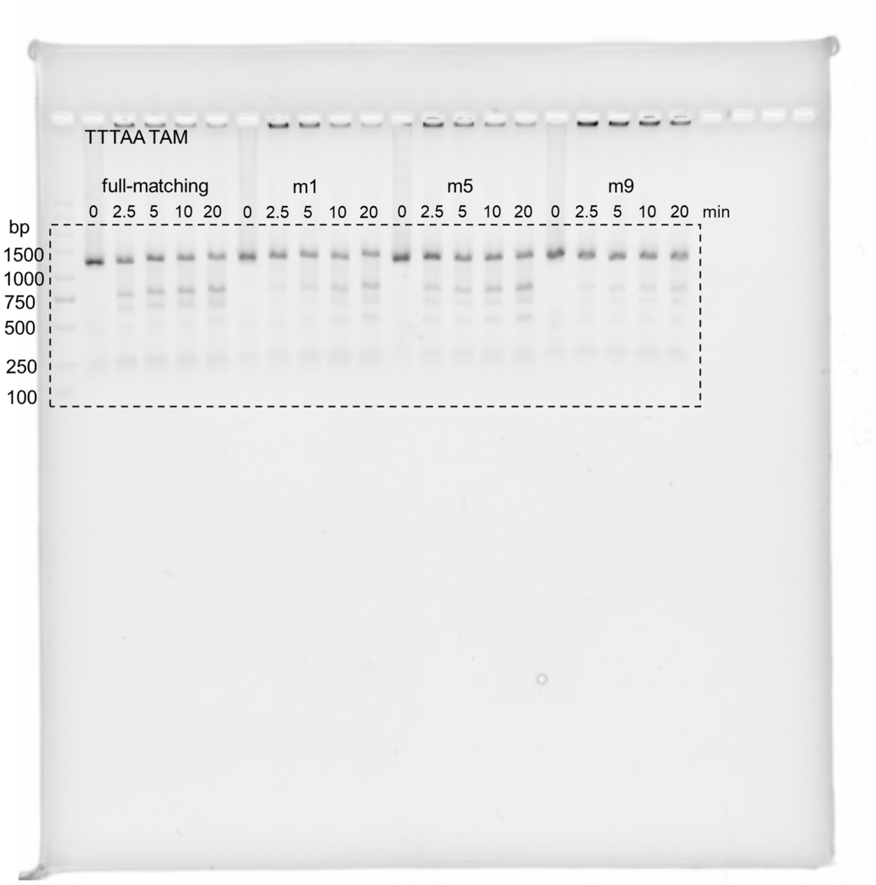

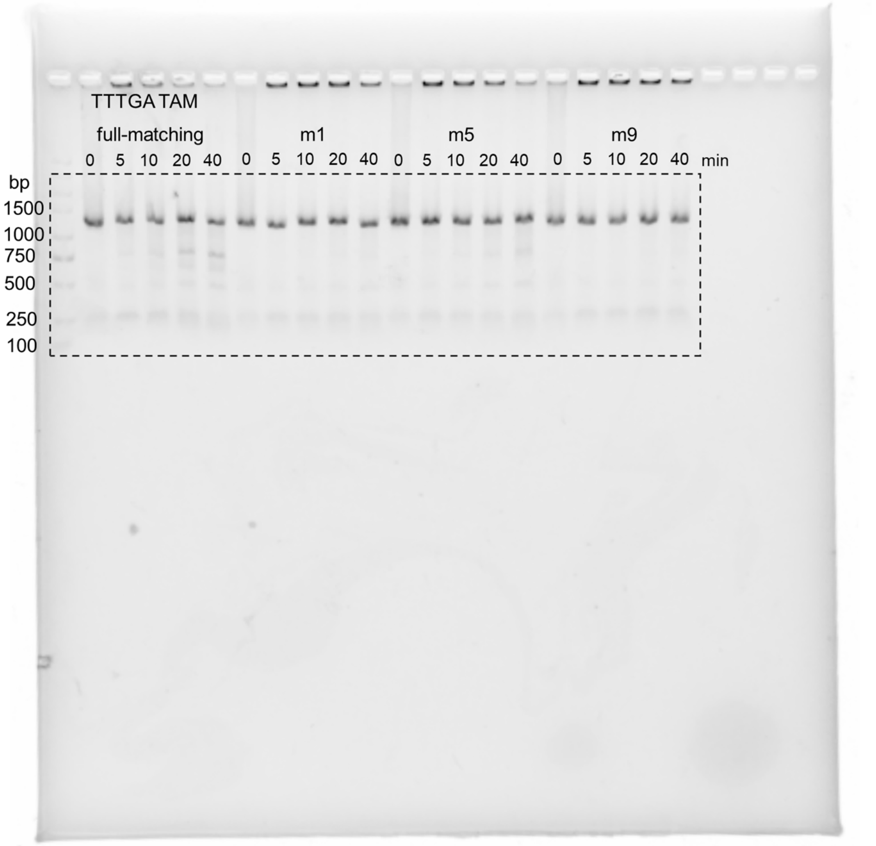

Source Data- Supplementary Figure 15

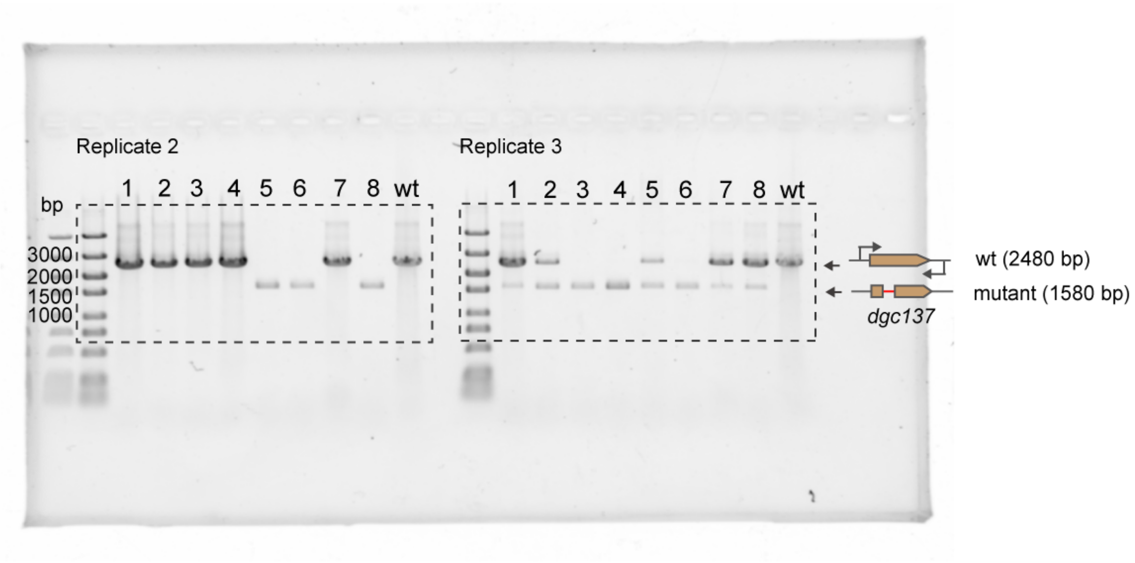

Source Data- Supplementary Figure 16

a

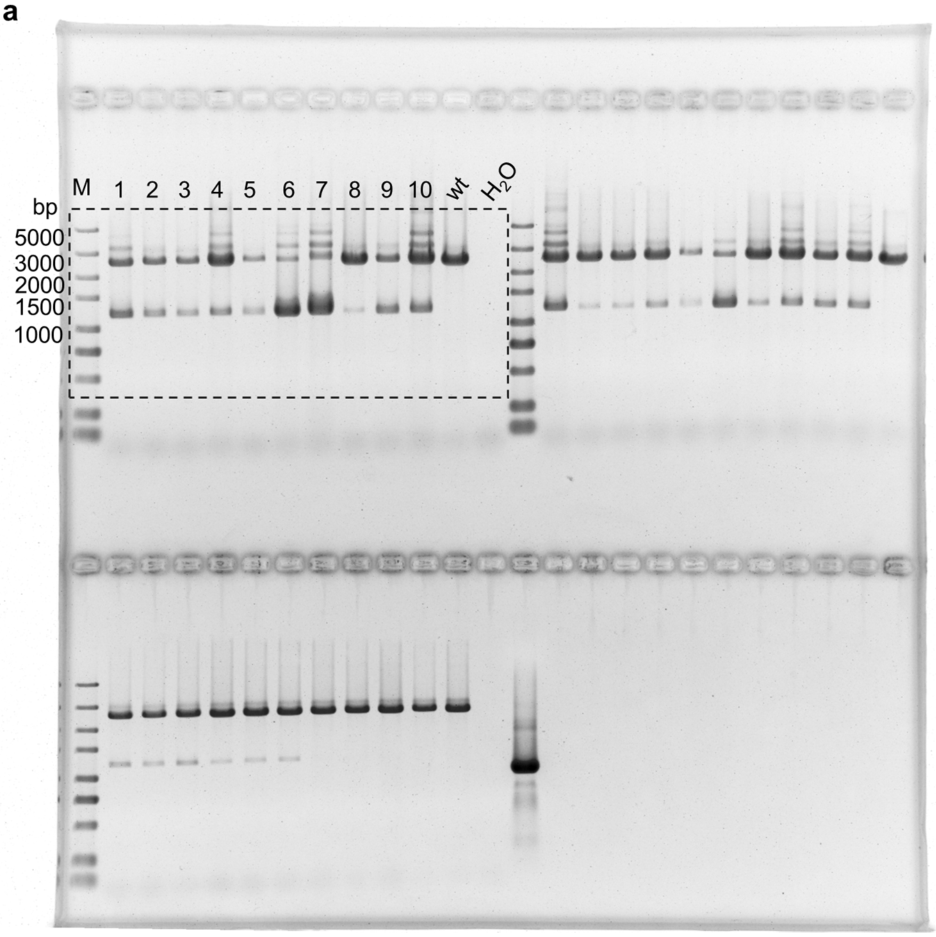

b

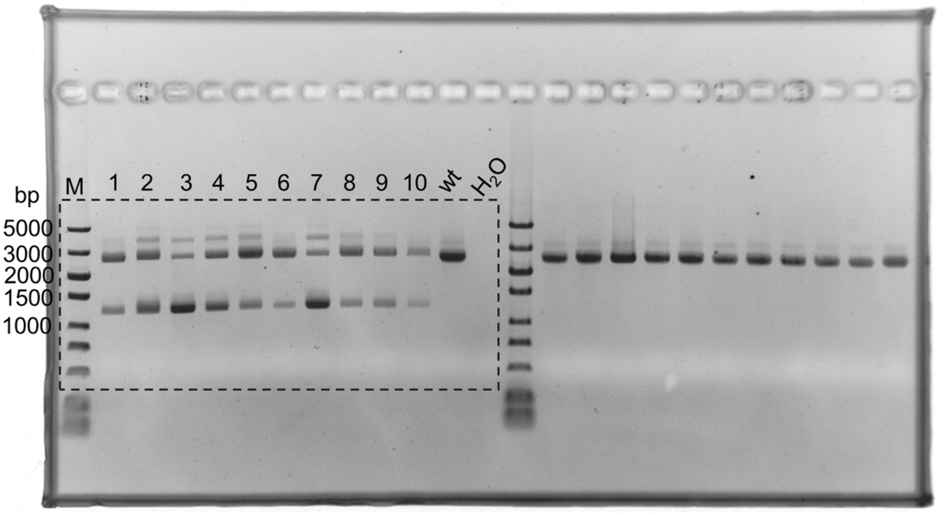

Supplement: Supplementary file 1 — Supplementary Information [file 41467_2024_47697_MOESM1_ESM.pdf]
