# Supplementary material for: Flexible TAM requirement of TnpB enables efficient single-nucleotide editing with expanded targeting scope
Source: Nat Commun. 2024 Apr 24;15:3464. doi: 10.1038/s41467-024-47697-4 (PMC11043419; doi:10.1038/s41467-024-47697-4)
Supplement: Supplementary file 4 — Description of Additional Supplementary Files [file 41467_2024_47697_MOESM4_ESM.pdf]

Title: Supplementary Data 1

Description: Information of strains, plasmids, oligos and target sequences.
