# Supplementary material for: Flexible TAM requirement of TnpB enables efficient single-nucleotide editing with expanded targeting scope
Source: Nat Commun. 2024 Apr 24;15:3464. doi: 10.1038/s41467-024-47697-4 (PMC11043419; doi:10.1038/s41467-024-47697-4)

Source data Fig. 6c

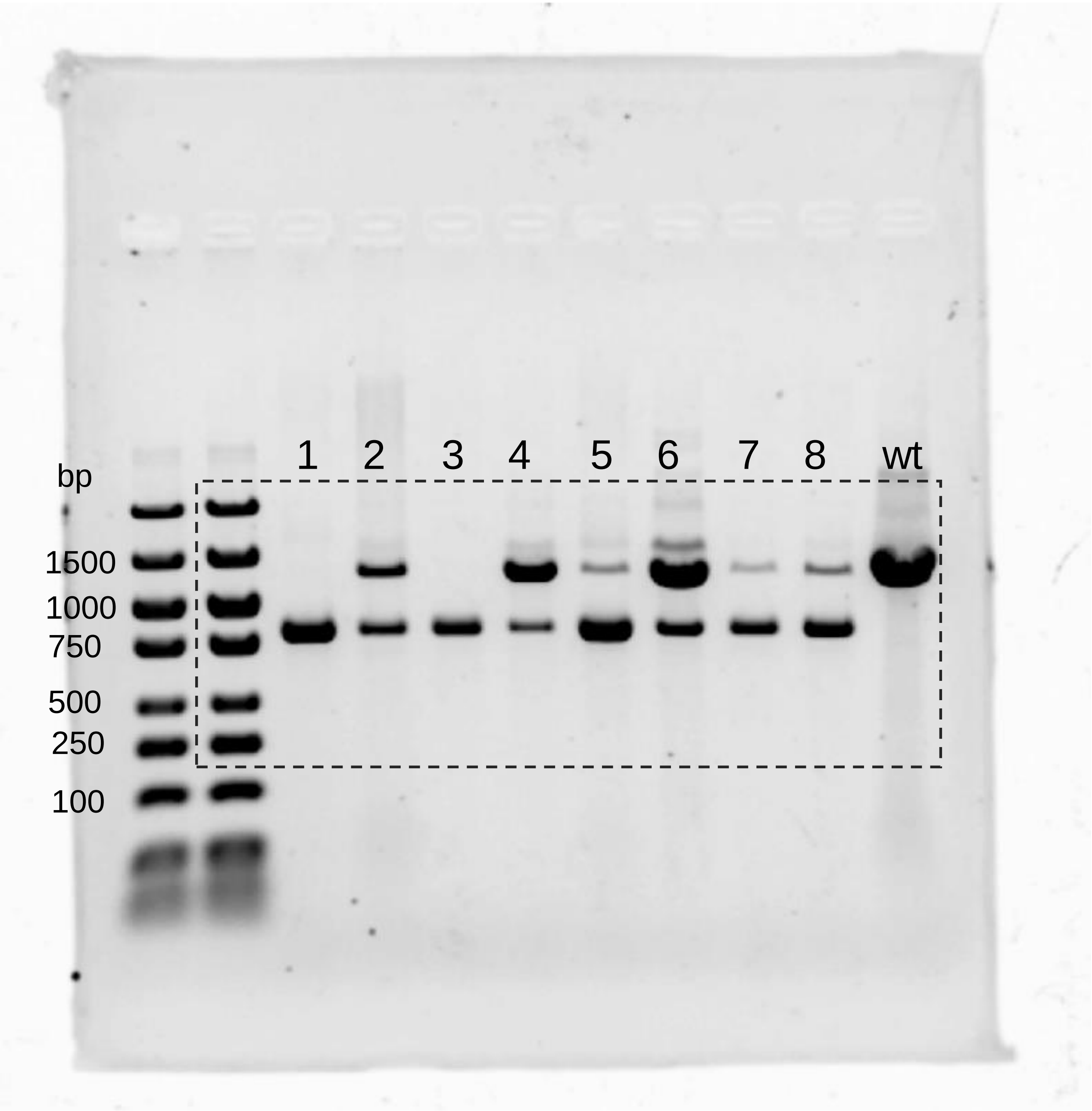

Supplement: Supplementary file 6 — Source Data file [file Fig.6 blot.pdf › 41467_2024_47697_MOESM6_ESM/Fig.6 blot.pdf]
